# Efficacy of soy protein concentrate replacing animal protein supplements in mucosa-associated microbiota, intestinal health, and growth performance of nursery pigs

**DOI:** 10.1016/j.aninu.2023.06.007

**Published:** 2023-06-30

**Authors:** Zixiao Deng, Marcos Elias Duarte, Sung Woo Kim

**Affiliations:** Department of Animal Science, North Carolina State University, Raleigh, NC 27695, USA

**Keywords:** Blood plasma, Fish meal, Intestinal health, Nursery pigs, Poultry meal, Soy protein concentrate

## Abstract

This study investigated the effects of using soy protein concentrate (SPC) to replace animal protein supplements on mucosa-associated microbiota, intestinal health, and growth performance of nursery pigs. Fifty-six newly weaned pigs (BW = 6.4 ± 0.6 kg) were allotted to 5 treatments in a randomized complete block design. Pigs were fed for 35 d in 3 phases (P; 1, 2, 3) for 10, 12, 13 d, respectively. Dietary treatments were: (1) basal diet with fish meal (P1: 4%, P2: 2%, and P3: 1%), poultry meal (P1: 10%, P2: 8%, and P3: 4%), and blood plasma (P1: 4%, P2: 2%, and P3: 1%), where SPC replacing none (NC); (2) basal diet with SPC replacing fish meal (RFM); (3) basal diet with SPC replacing poultry meal (RPM); (4) basal diet with SPC replacing blood plasma (RBP); and (5) basal diet with SPC replacing all animal protein supplements (PC). Growth performance was recorded for each phase. Pigs were euthanized on d 35 to collect jejunal mucosa and tissue to evaluate intestinal health and microbiota, and ileal digesta to measure apparent ileal digestibility (AID) of nutrients. Data were analyzed using the MIXED procedure of SAS. Overall, RFM, RPM, and RBP did not affect growth performance, whereas PC decreased (*P* < 0.05) ADG and ADFI. The RPM increased (*P* < 0.05) *Prevotella stercorea* and decreased (*P* < 0.05) *Helicobacter rappini*. The PC decreased (*P* < 0.05) *H. rappini*, whilst increasing (*P* < 0.05) *Prevotella copri*, *Propionibacterium acnes*, and *Pelomonas aquatica*. The RFM tended to increase (*P* = 0.096) immunoglobulin A in the jejunum. The PC tended to decrease (*P* = 0.078) jejunal crypt cell proliferation. There were no differences in the villus height, AID of nutrients, intestinal inflammation, and intestinal oxidative stress among treatments. In conclusion, SPC can replace fish meal, poultry meal, or blood plasma individually without affecting growth performance and intestinal health, and AID of nutrients of nursery pigs. Particularly SPC replacing poultry meal benefitted intestinal health by reducing *H. rappini* and increasing *P. stercorea*. However, SPC replacing all three animal protein supplements reduced growth of nursery pigs mainly by reducing feed intake.

## 1 Introduction

Weaning stress induced by environmental, social, and dietary changes is the most stressful event for nursery pigs, which can contribute to potential disorders in the intestine and immune system, reducing growth and health of pigs (Campbell et al., 2013; Moeser et al., 2007). In addition, the reduced weight gain immediately after weaning can extend the number of day to market (Kats et al., 1992). In order to mitigate these adverse effects from weaning stress, various feeding strategies utilizing different feedstuffs have been suggested for nursery pigs (Kim and Duarte, 2021; Pluske, 2013; Zheng et al., 2021).

Inclusion of high-quality protein supplements in feeds for nursery pigs are essential to reduce the negative impact of dietary changes during weaning. Animal protein supplements are widely used in feeds for nursery pigs because of their high nutrient digestibility and presence of functional proteins (Torrallardona, 2010). Fish meal is a typical protein supplement added in feeds for nursery pigs due to its high crude protein content and balanced amino acid profile, which can meet the nutritional requirements and improve feed intake of pigs (Bergström et al., 1997; Kim and Easter, 2001). Blood plasma has also been shown to improve post-weaning performance, possibly by enhancing intestinal immune status, intestinal barrier function, and intestinal morphology (Peace et al., 2011; Tran et al., 2014; Weaver et al., 2014). In addition, poultry meal is used as a protein supplement in diets of nursery pigs, due to the similar crude protein content and amino acid profiles compared to fish meal (Hicks and Verbeek, 2016; Keegan et al., 2004). However, price volatility, limited supply, and potential safety issues may limit the use of animal protein supplements in nursery diets (Kim et al., 2019).

Soybean meal (SBM) is a major co-product of soybean oil extraction, and is the most common plant protein supplement used in swine diets due to it amino acid profile similar to animal protein supplements and relatively stable supply (Banaszkiewicz, 2011). However, the presence of allergenic proteins (glycinin and β-conglycinin) in SBM limits its use in diets for nursery pigs (Taliercio and Kim, 2013; Wang et al., 2014).

Soy protein concentrate (SPC) is manufactured mainly via ethanol extraction of defatted soy flakes (Peisker, 2001). The manufacturing process reduces fibers, soluble carbohydrates, and the allergenicity of proteins but retains relatively high concentration of crude protein compared to SBM (Endres, 2001; Lusas and Riaz, 1995). In addition, ethanol extraction can remove some bitter off-flavors that may reduce feed intake (Morr and Ha, 1991). Previous study has shown that pigs fed SPC had a higher weight gain and feed efficiency than SBM (Zhang et al., 2013). Deng et al. (2022) has indicated that replacement of animal protein supplements with SPC in nursery diets can linearly decrease the diet cost when more SPC is included. In addition, the source of dietary protein can impact intestinal microbiota of pigs, because different protein sources could alter the luminal environment and substrate availability for microorganisms in the intestine (Duarte and Kim, 2022a; Rist et al., 2013). Previous studies reported that plant source proteins were more likely to promote the proliferation of beneficial bacteria compared to animal protein sources such as fish meal (Cao et al., 2016). Therefore, SPC could be considered a protein supplement to replace some high-cost and relatively short-supplied animal protein supplements in the nursery diet.

It was hypothesized that SPC can replace animal protein supplements without affecting intestinal health, nutrient digestibility, and growth performance of nursery pigs. The objective of current study was to investigate the effect of SPC replacing animal protein supplements on growth performance, nutrient digestibility, mucosa-associated microbiota, and intestinal health of nursery pigs.

## 2 Materials and methods

The experimental procedure was reviewed and approved by North Carolina State University Institutional Animal Care and Use Committee (Raleigh, NC) following the North Carolina State Animal Care and Use Procedures (REG 10.10.01). This experiment was conducted at the Metabolism Educational Unit of North Carolina State University (Raleigh, NC, USA).

### 2.1 Experimental design, animals, and diets

Fifty-six pigs (PIC Camborough × DNA 600, 28 barrows and 28 gilts) weaned at 21 d of age (initial body weight at 6.4 ± 0.6 kg) were obtained from Kilpatrick Hog Farm (Magnolia, NC, USA) which is a commercial farm. A randomized complete block design was used to allot the pigs using sex (barrow and gilt) and initial body weight (heavier or lighter than 6.5 kg body weight) as blocks. Within blocks, pigs were allotted to 5 dietary treatments consisting of a basal diet with animal protein supplements (SPC replacing none [NC], *n* = 12); basal diet with SPC (X-Soy 200, CJ Selecta, MG, Brazil) replacing fish meal (RFM, *n* = 12), basal diet with SPC replacing poultry meal (RPM, *n* = 12), basal diet with SPC replacing blood plasma (RBP, *n* = 12), or basal diet with SPC replacing all animal protein supplements (PC, *n* = 8). Compositions of nutrients and antinutritional factors in protein supplements used in the feeds are shown in Deng et al. (2022). NC dietary treatment contained 4%, 2%, 1% fish meal, 4%, 2%, 1% blood plasma, and 10%, 8%, 4% poultry meal in phases 1, 2, 3, respectively (Table 1). All nutrients in the experimental diets met or were slightly higher than the requirement levels suggested by NRC (2012). Pigs were fed experimental diets based on 3 phases for 35 d: phase 1 for 10 d (to 7 kg BW), phase 2 for 12 d (7 to 11 kg BW), and phase 3 for 13 d (11 to 20 kg BW). Pigs were housed in a single room and individually in pens (1.50 m × 0.74 m). Diets and water were supplied ad libitum. Titanium dioxide (0.4%) was supplemented to phase 3 diets during the last 5 d of the experiment as an indigestible external marker to evaluate the apparent ileal digestibility (AID) of nutrients. The Feed Mill Educational Unit at North Carolina State University produced all the experimental diets. The nutritional content of all experimental diets was sampled and submitted to the North Carolina Department of Agriculture and Consumer Services for analysis.

**Table 1:** The composition of experimental diets.^1^

Item	Phase 1					Phase 2					Phase 3				
	NC	PC	RFM	RPM	RBP	NC	PC	RFM	RPM	RBP	NC	PC	RFM	RPM	RBP
Feedstuff, % as-is basis															
Corn, yellow	28.99	26.68	27.65	31.62	25.61	40.67	40.10	39.89	42.59	38.92	62.57	62.31	62.22	63.52	61.66
Whey permeate	24.00	24.00	24.00	24.00	24.00	15.00	15.00	15.00	15.00	15.00	5.00	5.00	5.00	5.00	5.00
Soybean meal, 48% CP	16.00	16.00	16.00	16.00	16.00	19.00	19.00	19.00	19.00	19.00	23.00	23.00	23.00	23.00	23.00
Cookie meal	10.00	10.00	10.00	10.00	10.00	10.00	10.00	10.00	10.00	10.00	0.00	0.00	0.00	0.00	0.00
Poultry meal	10.00	0.00	10.00	0.00	10.00	8.00	0.00	8.00	0.00	8.00	4.00	0.00	4.00	0.00	4.00
Fish meal	4.00	0.00	0.00	4.00	4.00	2.00	0.00	0.00	2.00	2.00	1.00	0.00	0.00	1.00	1.00
Blood plasma	4.00	0.00	4.00	4.00	0.00	2.00	0.00	2.00	2.00	0.00	1.00	0.00	1.00	1.00	0.00
SPC^2^	0.00	18.50	5.00	6.00	7.50	0.00	11.05	2.50	4.80	3.75	0.00	5.55	1.25	2.40	1.90
Poultry fat	1.00	1.70	1.00	1.70	1.00	1.00	1.60	1.00	1.60	1.00	1.00	1.30	1.00	1.30	1.00
L-Lys HCl	0.52	0.55	0.53	0.52	0.54	0.51	0.53	0.51	0.51	0.52	0.42	0.42	0.42	0.42	0.42
L-Met	0.25	0.24	0.25	0.22	0.26	0.21	0.20	0.21	0.19	0.22	0.14	0.14	0.14	0.14	0.15
L-Thr	0.17	0.03	0.12	0.13	0.12	0.15	0.08	0.13	0.12	0.13	0.12	0.08	0.12	0.12	0.12
L-Trp	0.02	0.00	0.00	0.01	0.02	0.01	0.00	0.01	0.00	0.01	0.00	0.00	0.00	0.00	0.00
L-Val	0.00	0.00	0.00	0.05	0.00	0.00	0.01	0.00	0.04	0.00	0.00	0.00	0.00	0.00	0.00
Limestone, ground	0.40	0.80	0.60	0.85	0.30	0.60	0.83	0.70	0.85	0.60	0.80	0.90	0.80	0.80	0.80
Dicalcium phosphate	0.00	0.85	0.20	0.25	0.00	0.20	0.95	0.40	0.65	0.20	0.55	0.90	0.65	0.90	0.55
Zinc oxide^3^	0.25	0.25	0.25	0.25	0.25	0.25	0.25	0.25	0.25	0.25	0.00	0.00	0.00	0.00	0.00
Salt	0.22	0.22	0.22	0.22	0.22	0.22	0.22	0.22	0.22	0.22	0.22	0.22	0.22	0.22	0.22
Mineral premix^4^	0.15	0.15	0.15	0.15	0.15	0.15	0.15	0.15	0.15	0.15	0.15	0.15	0.15	0.15	0.15
Vitamin premix^5^	0.03	0.03	0.03	0.03	0.03	0.03	0.03	0.03	0.03	0.03	0.03	0.03	0.03	0.03	0.03
Calculated composition, % as-is basis															
DM	91.2	91.6	91.4	91.1	91.6	90.7	90.8	90.8	90.5	90.8	89.7	89.8	89.7	89.6	89.8
ME, kcal/kg	3,436	3,431	3,434	3,438	3,440	3,419	3,414	3,415	3,418	3,420	3,372	3,372	3,372	3,372	3,373
Crude protein	24.6	23.6	25.0	22.0	25.8	22.6	21.3	22.8	20.5	23.2	20.9	20.2	21.0	19.8	21.2
SID^6^ Lys	1.50	1.50	1.50	1.50	1.50	1.35	1.35	1.35	1.35	1.35	1.23	1.23	1.23	1.23	1.23
SID Met + Cys	0.82	0.82	0.82	0.82	0.82	0.74	0.74	0.74	0.74	0.74	0.68	0.68	0.68	0.69	0.68
SID Trp	0.25	0.26	0.25	0.25	0.25	0.22	0.23	0.23	0.22	0.22	0.20	0.21	0.21	0.20	0.20
SID Thr	0.88	0.88	0.88	0.88	0.88	0.79	0.79	0.79	0.79	0.79	0.74	0.73	0.74	0.74	0.74
Ca	0.88	0.85	0.85	0.85	0.87	0.80	0.80	0.81	0.80	0.81	0.71	0.70	0.70	0.70	0.71
STTD P^7^	0.50	0.45	0.46	0.45	0.48	0.41	0.40	0.40	0.40	0.40	0.33	0.33	0.33	0.33	0.33
Total P	0.73	0.67	0.69	0.64	0.73	0.64	0.63	0.64	0.61	0.64	0.58	0.57	0.58	0.57	0.58
Glycinin,^8^ mg/kg	18.0	18.0	18.0	18.0	18.0	21.4	21.4	21.4	21.4	21.4	25.9	25.9	25.9	25.9	25.9
β-Conglycinin,^9^ mg/kg	20.0	20.0	20.0	20.0	20.0	23.8	23.8	23.8	23.8	23.8	28.8	28.8	28.8	28.8	28.8
Analyzed composition, % as-is basis															
DM	91.8	91.7	91.4	91.0	91.8	90.3	90.5	90.3	90.0	90.9	88.9	88.8	88.8	88.2	89.0
Crude ash	6.76	6.75	6.56	6.59	6.60	6.25	6.37	6.22	6.19	6.54	4.74	4.88	4.37	4.66	4.88
CP	24.7	22.5	24.3	21.1	25.3	22.1	20.7	22.8	19.7	23.1	20.7	19.7	19.1	18.9	20.8
Neutral detergent fiber	6.80	6.08	7.09	5.42	7.57	7.36	6.64	7.82	6.82	7.88	6.42	7.09	7.21	7.14	7.90
Acid detergent fiber	3.09	3.48	3.2	2.89	3.45	3.58	3.87	3.63	3.26	3.69	2.94	3.22	2.85	3.07	3.46
Ca	0.91	0.78	0.75	0.81	0.79	0.80	0.83	0.77	0.81	0.89	0.76	0.73	0.76	0.77	0.82
Total P	0.77	0.65	0.68	0.63	0.71	0.67	0.66	0.69	0.66	0.69	0.55	0.55	0.48	0.56	0.58

SPC = soy protein concentrate; DM = dry matter.

1 NC, PC, RFM, RPM, and RBP: basal diet without SPC; basal diet with SPC replacing all animal protein supplements; basal diet with SPC replacing fish meal; basal diet with SPC replacing poultry meal; basal diet with SPC replacing blood plasma.

2 SPC (X-Soy 200, Selecta, MG, Brazil).

3 Zinc oxide was targeted to 2,500 mg/kg in phase 1 and phase 2 diets.

4 The trace mineral premix provided per kilogram of complete diet: 33 mg of Mn as manganous oxide, 110 mg of Fe as ferrous sulfate, 110 mg of Zn as zinc sulfate, 16.5 mg of Cu as copper sulfate, 0.30 mg of I as ethylenediamine dihydroiodide, and 0.30 mg of Se as sodium selenite.

5 The vitamin premix provided per kilogram of complete diet: 6,614 IU of vitamin A as vitamin A acetate, 992 IU of vitamin D_3_, 19.8 IU of vitamin E, 2.64 mg of vitamin K as menadione sodium bisulfate, 0.03 mg of vitamin B_12_, 4.63 mg of riboflavin, 18.52 mg of D-pantothenic acid as calcium pantothenate, 24.96 mg of niacin, and 0.07 mg of biotin.

6 SID, standardized ileal digestibility.

7 STTD P, standardized total tract digestible phosphorus.

8 The concentration of glycinin in the diets was calculated based on the analyzed glycinin of soybean meal and soy protein concentrate.

9 The concentration of β-conglycinin in the diets was calculated based on the analyzed β-conglycinin of soybean meal and soy protein concentrate.

### 2.2 Growth performance and fecal score

The average BW, ADG, ADFI, and G:F were calculated based on the recorded BW and feed intake at the end of each phase. Fecal scores were recorded daily by the same person based on 1 to 5 scales: (1) very  hard and dry stool, (2) firm stool, (3) normal stool, (4) loose stool, and (5) watery stool with no shape following Weaver and Kim (2014).

### 2.3 Samples collection

At the end of experiment, pigs were euthanized by exsanguination after penetration of a captive bolt to the head. The gastrointestinal tract was removed for collection of samples. Ileal digesta was collected from 50 cm anterior to the ileocecal junction in a 100-mL container and put on the ice, then stored at −20 °C for measurement of AID of nutrients. To evaluate morphology, 5 cm of mid-jejunal tissue (3 m immediately after the duodenojejunal junction) were collected in a 50-mL falcon tube with 10% buffered formaldehyde. Another section of mid-jejunum (15 cm) was rinsed with sterile saline solution (0.9%) to remove the digesta content. The section was opened, and the mucosa samples were collected into 2-mL tubes by scrapping using a microscope slide, then the tubes were immediately snap frozen in liquid nitrogen and stored at −80 °C for immune and oxidative stress measurements and mucosa-associated microbiota analysis.

### 2.4 Relative abundance and diversity of jejunal mucosa-associated microbiota

Mucosa samples without digesta content were used to analyze diversity and relative abundance of mucosa-associated microbiota as previously reported (Duarte et al., 2021; Moita et al., 2021; Sun et al., 2021). The deoxyribonucleic acid (DNA) extracted from mid-jejunal mucosa (approximately 200 mg) was used for measurement of microbiota. The DNA was extracted by using DNA Stool Mini Kit (#51604, Qiagen; Germantown, MD, USA). The weighed samples were mixed with 1 mL InhibitEX buffer in 2 mL microcentrifuge tubes and vortexed until the samples were homogenized, then centrifuged at 13,000 × *g* for 1 min. The supernatant (600 μL) was transferred in a new 2 mL microcentrifuge tube and mixed with 25 μL proteinase K and 600 μL Buffer AL, then vortexed the mixture for 15 s. The mixture was placed in a water bath for incubation at 70 °C for 10 min. Next, 600 μL ethanol (96% to 100%) was added to the lysate and vortexed, then transferred 600 μL to the QIAamp spin column for centrifuging at 13,000 × *g* for 1 min. Buffer AW1 (500 μL) and AW2 (500 μL) were separately added in the QIAamp spin column for washing at 13,000 × *g* for 1 min. At the end, 200 μL Buffer ATE was added in QIAamp spin column to elute DNA at 13,000 × *g* for 1 min. The collected DNA was transported to Mako Medical Laboratories (Raleigh, NC, USA) for 16S rRNA microbiome sequencing analysis. Samples were first created using Ion Chef equipment as a template, and then analyzed using an Ion S5 system (Thermo Fisher Scientific, Wilmington, DE, USA). Ion 16S Metagenomics Kit 113 (Thermo Fisher Scientific) was used to amplify various sequences V2, V3, V4, V6, V7, V8, and V9. These sequences were then processed using Torrent Suite Software (version 5.2.2, Thermo Fisher Scientific) to get raw unaligned sequence data files. Then Ion Reporter Software Suite (version 5.2.2) of bioinformatics analysis tools (Thermo Fisher Scientific) were used to process microbial analysis including alignment to GreenGenes and MicroSeq databases, alpha and beta diversity plot generation, and operational taxonomic unit (OTU) table generation. Finally, sample analyses were carried out utilizing the Ion Reporter's Metagenomics 16S workflow powered by QIIME (version w1.1). The results of relative abundance were calculated based on OTU table and the results of alpha diversity were calculated based on alpha diversity plot (The OTU with the relative abundance <0.5% were considered as others).

### 2.5 Immune and oxidative stress status

The sample of jejunal mucosa (1 g) was weighed and then suspended with 1 mL phosphate-buffered saline (PBS, 0.01 mol/L phosphate, 0.0027 mol/L KCl, and 0.137 mol/L NaCl) on the ice. The samples were homogenized using a tissue homogenizer (Tissuemiser; Thermo Fisher Scientific). Following Holanda and Kim (2021), the homogenized samples were transferred into new microcentrifuge tube and centrifuged at 13,000 × *g* for 10 min. The supernatant (100 μL) was transferred into 6 aliquots individually and stored at −80 °C.

The commercial kits were used to measure the total protein concentration of jejunal mucosa, tumor necrosis factor alpha (TNF-α), immunoglobulin G (IgG), immunoglobulin A (IgA), interleukin 8 (IL-8), malondialdehyde (MDA), and protein carbonyl based on the instruction manual. The ELISA plate reader (Synergy HT, BioTek Instruments, Winooski, VT, USA) and software (Gen5 Data Analysis Software, BioTek Instruments) were used to determine the OD value. The corresponding concentrations were calculated based on the instruction manual and absorbance of standard curves.

The Pierce BCA Protein Assay Kit (#23225, Thermo Fisher Scientific) was used to measure the total protein concentration as described by Holanda et al. (2020). To get the appropriate range of measurement, the homogenized mucosal supernatant was diluted (1:60) with PBS. Samples and standards (25 μL) were added to microplate wells, followed by 200 μL of working reagent. The plate was then incubated at 37 °C for 30 min. The absorbance was measured at 562 nm wavelength. The total protein concentration was further used to normalize the concentration of other mucosal measurements.

The Porcine TNF-α Immunoassay Kit (#PTA00, R&D Systems; Minneapolis, MN, USA) was used to measure TNF-α concentration as described by Cheng et al. (2021). First, 100 μL of both the samples and standards were added to individual wells on the plate, followed by the addition of 100 μL of detection antibody. The plate was then incubated at room temperature for 2 h. Next, the plate was consecutively treated with 100 μL of Streptavidin-HRP and 100 μL of substrate solution for analysis. Finally, the reaction was stopped with 50 μL of stop solution. The absorbance was measured at 450 nm wavelength and corrected at 570 nm wavelength. The concentration of TNF-α was expressed as pg/mL protein. The Porcine IL-8/CXCL8 DuoSet ELISA kit (#DY535, R&D Systems) was used to measure the concentration of IL-8 as described by Cheng et al. (2021). To get the appropriate range of measurement, all samples were diluted (1:5) with reagent diluent before measurement. After adding 100 μL of samples and standards to each well of the plate, 100 μL of detection antibody was added and the plate was incubated for 2 h at room temperature. The analysis was carried out by consecutively adding 100 μL of Streptavidin-HRP and 100 μL of substrate solution. Finally, the reaction was halted using 50 μL of stop solution. The absorbance was measured at 450 nm wavelength and corrected at 570 nm wavelength. The concentration of IL-8 was expressed as pg/mL protein.

The ELISA kits (E101-102 and E101-104, Bethyl Laboratories, Montgomery, TX, USA) were used to measure the concentration of IgA and IgG as described by Holanda et al. (2020). To get the appropriate range of measurement, the mucosal supernatants were diluted (1:1,200 and 1:2,400, respectively) with PBS. Samples and standards were added to each well of the plate and incubated at room temperature for 1 h. Next, 100 μL of anti-IgA detection antibody was added and the plate was further incubated at room temperature for 1 h. The analysis was carried out by sequentially adding 100 μL of HRP solution and 100 μL of 3,3′,5,5′-tetramethylbenzidine (TMB) substrate solution. Finally, the reaction was stopped by adding 100 μL of stop solution. The absorbance was measured at 450 nm wavelength and the concentration of IgA and IgG were expressed as μg/mg of protein.

The OxiSelect TBARS MDA Quantitation Assay Kit (#STA-330, Cell Biolabs, San Diego, CA, USA) was used to measure the concentration of MDA in mucosa as described by Moita et al. (2021). Samples and standards (100 μL) were added to separate 2 mL microcentrifuge tubes, followed by the addition of 100 μL of SDS lysis solution and 250 μL of thiobarbituric acid (TBA) reagent. The tubes were then placed in a water bath at 95 °C for 1 h. After cooling, the tubes were centrifuged at 3,000 × *g* for 15 min to obtain the supernatant. The absorbance was measured at 532 nm wavelength. The concentration of MDA was expressed as nmol/mg protein.

The OxiSelect Protein Carbonyl ELISA Kit (#STA-310, Cell Biolabs) was used to measure the concentration of protein carbonyl as described by Moita et al. (2021). All supernatants were diluted in PBS to get 10 μg/mL before measurement and then all processes were conducted following the manufacturer's protocol. The diluted samples and standards were incubated overnight at 4 °C in each well. After washing, DNPH working solution (100 μL) was added and incubated for 45 min at room temperature. Then, the plate was washed and incubated with blocking solution (200 μL) for 1.5 h on an orbital shaker. The plate was washed again before adding anti-DNP antibody, which was incubated for 1 h. After washing, HRP-conjugated secondary antibody was added, and the plate was incubated for 1 h. Substrate solution (100 μL) was added, followed by incubation for 5 min at room temperature. The reaction was stopped using stop solution (100 μL). The absorbance was measured at 450 nm wavelength and the concentration of protein carbonyl was described as nmol/mg protein.

### 2.6 Intestinal morphology and crypt cell proliferation

Two segments of mid jejunum per pig were fixed in 10% buffered formaldehyde for 48 h and then were transferred to a 70% ethanol solution. The dehydration, embedment, staining and Ki-67 assay of processed samples were processed at North Carolina State University Histology Laboratory (College of Veterinary Medicine, Raleigh, NC, USA). Biocare Intellipath stainer (Biocare Medical, Pacheco, CA, USA) was used for automated Ki-67 staining. Primary monoclonal antibody of Ki-67 (#ACR325, Biocare Medical) was diluted (1:100) and then incubate for 30 min with samples. Vector ImmPress Rabbit polymer was used for detection. Diaminobenzamine was used for staining as a chromogen. The microscope Olympus CX31 (Lumenera Corporation, Ottawa, ON, CAN) was used to measure villus height, villus width, and crypt depth. The pictures were taken at magnification 40× using a digital camera (Infinity 2-2 digital CCD, Lumenera, Ottawa, ON, Canada) and analyzed with a software (Infinity Analyze microscopy software, Lumenera). Ten intact villi and associated crypts in each slide were measured as described by Cheng et al. (2021). The villus length was measured from the junction of villi and crypt to the top of the villi; the villus width was measured at the middle of the villi; the crypt depth was measured from the bottom of the crypt to the junction of villi and crypt. The villus height to crypt depth (VH:CD) ratio was calculated using the villus height divided by the crypt depth. The pictures of crypts were taken by microscope Olympus CX31 at 100× to calculate the proportion of Ki-67 positive cells as indicator of proliferating cells in the crypt. The cropped pictures were processed by Image JS tool and analyzed by the same person.

### 2.7 Apparent ileal digestibility

Ileal digesta samples were freeze-dried for 48 h with a freeze dryer (24D 48, Virtis, Gardiner, NY, USA). The dried digesta and diet samples were used to measure the dry matter (DM) and ether extract (EE) based on method (930.15) and method (2003.06) of AOAC (2007). A bomb calorimeter (Model 6200, Parr Instrument Company, Moline, IL, USA) was used to measure gross energy (GE) in feed and digesta samples. The composition of crude protein (CP) and amino acids (AA) in diet and digesta samples were sent to Experiment Station Chemical Laboratories of University of Missouri-Columbia for analysis. The concentration of titanium dioxide in the diet and digesta were measured based on the approach as described by Myers et al. (2004). To calculate the AID of DM, GE, EE, CP, and AA, the function following Chen et al. (2020) was used:

AID (%) = {1 − [(TiO_2diet_/TiO_2digesta_) × (Nutrient_digesta_/Nutrient_diet_)]} × 100,

in which TiO_2diet_ and TiO_2digesta_ were the measured concentration of titanium dioxide in the feed and in the digesta; Nutrient_digesta_ and Nutrient_diet_ were the measured concentration of nutrient in the digesta and in the diet.

### 2.8 Statistical analysis

The MIXED procedure of SAS 9.4 (SAS Inc., Cary, NC, USA) was used to analyze the data. Experimental unit was the pig that was fed and housed individually. Main effect was dietary treatments. Dietary treatments were considered fixed effects and initial BW and sex were considered random effects. The means were calculated using the LSMEANS statement to ensure an unbiased estimation of the treatment means considering the unbalanced number of observations among treatments. Pre-planned contrasts were used to evaluate the effects of SPC supplementation replacing all animal protein supplements (NC vs. PC) and SPC replacing each animal protein supplement (NC vs. RFM, NC vs. RPM, and NC vs. RBP). It was considered statistical significance as *P*-value less than 0.05 and tendency as *P*-value between 0.05 and 0.10.

## 3 Results

### 3.1 Growth performance and fecal score

**Fig. 1:**
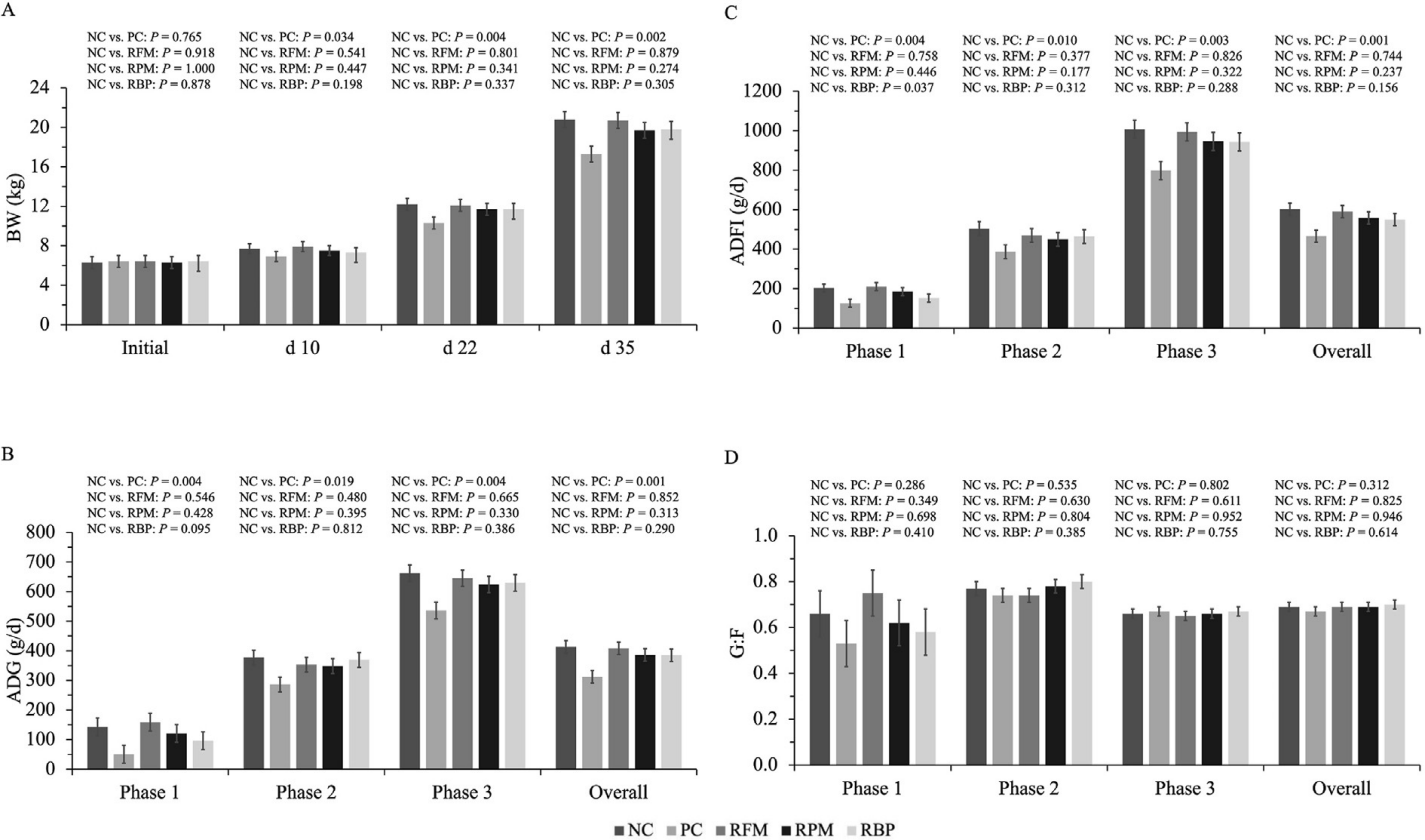
Growth performance of nursery pigs fed diets with SPC replacing all animal protein supplements, fish meal, poultry meal, and blood plasma. (A) Body weight of pigs at different time points. (B) Average daily gain of pigs during different phase and overall. (C) Average daily feed intake of pigs during different phase and overall. (D) Feed efficiency of pigs during different phase and overall. Values are presented as mean ± standard error of the mean. Dietary treatments were supplemented with SPC (X-Soy 200, Selecta, MG, Brazil) replacing none (NC), all animal protein supplements (PC), fish meal (RFM), poultry meal (RPM), or blood plasma (RBP) in nursery diets. SPC = soy protein concentrate.

**Fig. 2:**
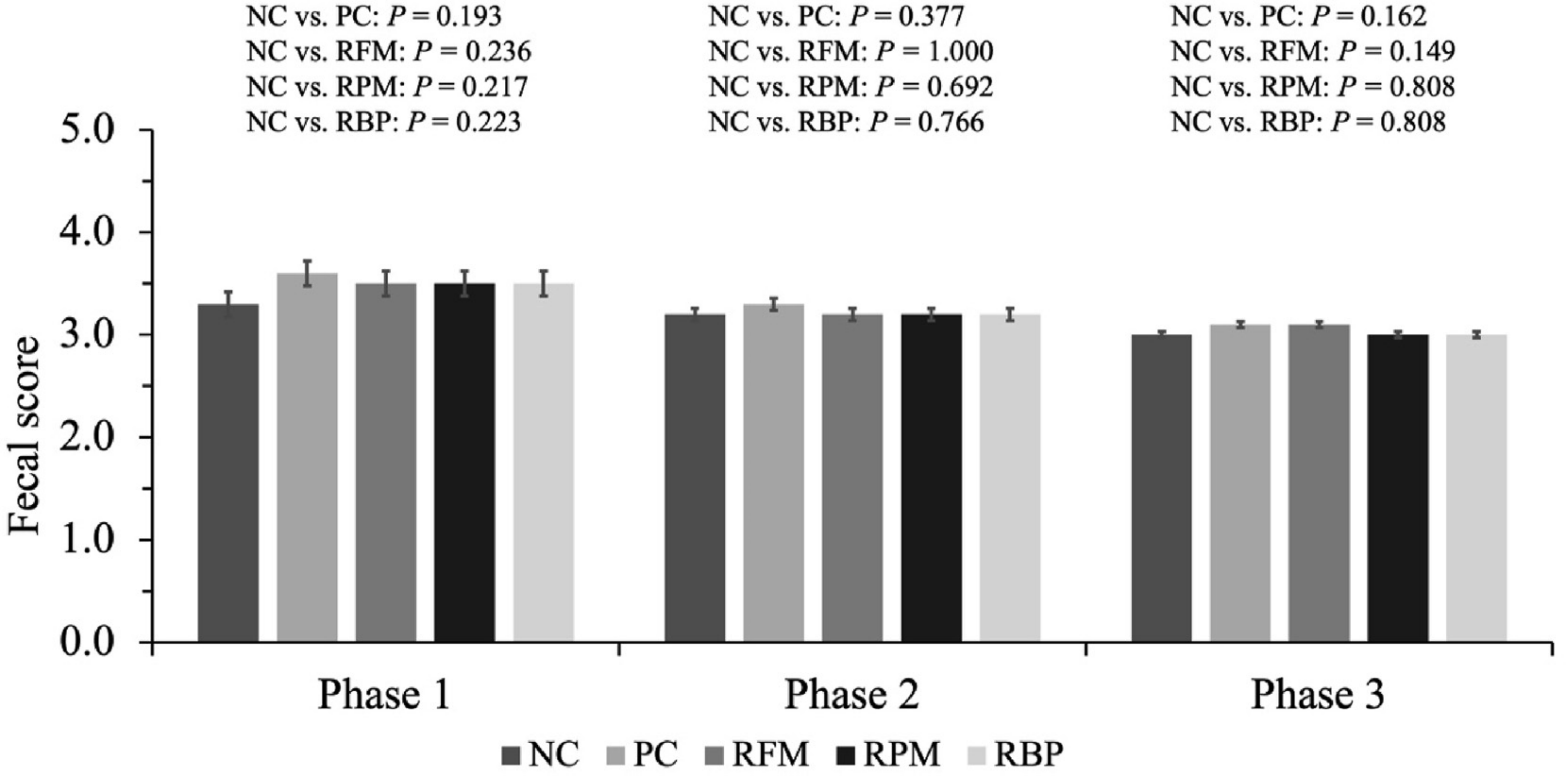
Fecal score of nursery pigs fed diets with SPC replacing fish meal, poultry meal, and blood plasma. Values are presented as mean ± standard error of the mean. Dietary treatments were supplemented with SPC (X-Soy 200, Selecta, MG, Brazil) replacing none (NC), all animal protein supplements (PC), fish meal (RFM), poultry meal (RPM), or blood plasma (RBP) in nursery diets. SPC = soy protein concentrate.

When SPC replaced blood plasma in the nursery diet, pigs decreased (*P* < 0.05) ADFI and tended to decrease (*P* = 0.095) ADG in P1 compared to NC (Fig. 1). When SPC replaced all animal protein supplements in the nursery diet, pigs decreased (*P* < 0.05) ADFI and ADG in entire experiment. Fecal scores were not affected by SPC replacing animal protein supplements on the entire experimental period (Fig. 2).

### 3.2 Relative abundance and diversity of jejunal mucosa-associated microbiota

**Fig. 3:**
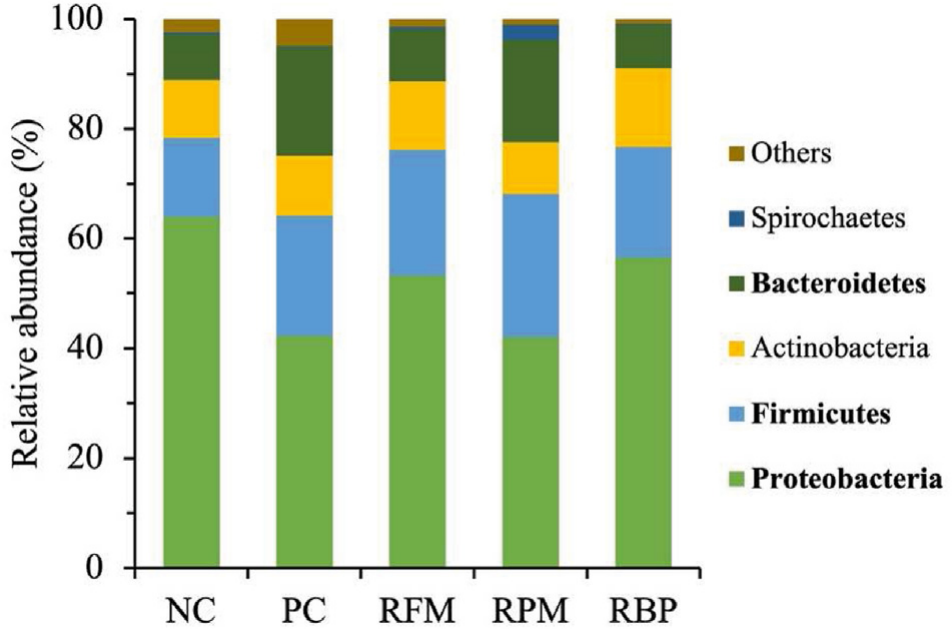
Relative abundance of jejunal mucosa-associated microbiota at phylum level in nursery pigs fed diets with SPC replacing fish meal, poultry meal, and blood plasma. Rach pattern represents a specific bacterial phylum. The relative abundance of phylum that did not achieve 0.5% were considered ‘Others’. Dietary treatments were supplemented with SPC (X-Soy 200, Selecta, MG, Brazil) replacing none (NC), all animal protein supplements (PC), fish meal (RFM), poultry meal (RPM), or blood plasma (RBP) in nursery diets. Proteobacteria: NC vs. PC (*P* < 0.05), NC vs. RPM (*P* < 0.05). Firmicutes: NC vs. RPM (*P* = 0.064). Bacteroidetes: NC vs. PC (*P* = 0.087), NC vs. RPM (*P* = 0.089). SPC = soy protein concentrate.

At phylum level (Fig. 3), when SPC replaced poultry meal in the nursery diets, pigs tended to increase the relative abundance of Firmicutes (*P* = 0.064) and Bacteroidetes (*P* = 0.089), and decreased (*P* < 0.05) the relative abundance of Proteobacteria. When SPC replaced all animal protein supplements in the nursery diet, pigs tended to increase (*P* = 0.087) the relative abundance of Bacteroidetes and decreased (*P* < 0.05) the relative abundance of Proteobacteria.

At family level (Table 2), when SPC replace fish meal in the nursery diet, pigs tended to decrease (*P* = 0.076) the relative abundance of Moraxellaceae. When SPC replaced poultry meal in the nursery diet, pigs tended to increase the relative abundance of Prevotellaceae (*P* = 0.090) and Micrococcaceae (*P* = 0.055), however it decreased (*P* < 0.05) the relative abundance of Helicobacteraceae. When SPC replaced blood plasma in the nursery diet, pigs tended to increase the relative abundance of Burkholderiaceae (*P* = 0.055) and Ruminococcaceae (*P* = 0.095) but tended to decrease (*P* = 0.077) the relative abundance of Moraxellaceae. When SPC replaced all animal protein supplements in the nursery diet, pigs increased (*P* < 0.05) the relative abundance of Enterobacteriaceae and Lactobacillaceae, and tended to increase the relative abundance of Prevotellaceae (*P* = 0.073), Propionibacteriaceae (*P* = 0.057), Lachnospiraceae (*P* = 0.079), Succinivibrionaceae (*P* = 0.088), and Streptococcaceae (*P* = 0.099), however it decreased (*P* < 0.05) the relative abundance of Helicobacteraceae, Moraxellaceae, Sphingomonadaceae, Aerococcaceae, and Bradyrhizobiaceae, and tended to decrease (*P* = 0.099) the relative abundance of Campylobacteraceae.

**Table 2:** Relative abundance of jejunal mucosa-associated microbiota at family level in nursery pigs fed diets with SPC replacing fish meal, poultry meal, and blood plasma.

Item	NC^1^	PC^1^	RFM^1^	RPM^1^	RBP^1^	SEM	*P*-value			
							NC vs. PC	NC vs. RFM	NC vs. RPM	NC vs. RBP
Helicobacteraceae	27.95	3.02	22.56	11.02	17.83	6.12	0.010	0.518	0.046	0.227
Prevotellaceae	7.48	18.94	7.24	17.16	7.08	6.79	0.073	0.965	0.090	0.942
Comamonadaceae	7.15	13.04	8.03	8.13	14.11	3.92	0.235	0.843	0.826	0.119
Moraxellaceae	6.43	0.01	1.71	3.05	1.72	1.92	0.032	0.076	0.200	0.077
Alcaligenaceae	3.92	<0.01	0.65	0.32	1.29	2.25	0.187	0.217	0.176	0.321
Clostridiaceae	3.68	4.61	5.35	5.21	2.88	3.10	0.822	0.653	0.682	0.830
Campylobacteraceae	2.95	0.13	1.31	2.59	0.97	1.13	0.099	0.279	0.814	0.192
Corynebacteriaceae	2.91	<0.01	1.71	1.90	2.51	0.68	0.007	0.200	0.279	0.661
Staphylococcaceae	2.78	0.06	2.94	5.99	4.56	1.86	0.330	0.949	0.200	0.474
Veillonellaceae	2.45	3.50	3.41	4.86	2.28	1.11	0.532	0.527	0.114	0.907
Microbacteriaceae	2.39	3.35	2.45	2.83	4.48	1.39	0.604	0.970	0.791	0.214
Sphingomonadaceae	1.95	0.17	1.73	1.63	2.00	0.58	0.048	0.777	0.688	0.949
Burkholderiaceae	1.79	0.78	2.15	1.85	3.54	0.77	0.315	0.691	0.945	0.055
Pseudomonadaceae	1.79	6.64	3.29	1.24	1.66	2.63	0.168	0.630	0.860	0.966
Propionibacteriaceae	1.51	5.20	3.27	1.00	2.14	1.68	0.057	0.303	0.765	0.712
Xanthomonadaceae	1.40	0.15	3.46	0.64	1.01	1.70	0.594	0.329	0.714	0.851
Enterobacteriaceae	1.34	13.41	1.81	2.34	4.03	3.47	0.020	0.916	0.823	0.552
Aerococcaceae	1.05	<0.01	0.49	0.55	0.34	0.31	0.032	0.192	0.246	0.102
Lactobacillaceae	1.04	8.15	4.22	4.07	3.17	1.90	0.017	0.224	0.247	0.413
Bifidobacteriaceae	1.02	2.05	1.69	0.97	1.71	0.61	0.270	0.422	0.958	0.411
Methylobacteriaceae	0.85	0.58	1.34	1.74	1.66	0.54	0.689	0.413	0.142	0.179
Lachnospiraceae	0.75	1.80	1.02	1.37	0.75	0.48	0.079	0.604	0.240	0.996
Succinivibrionaceae	0.70	2.76	0.69	1.71	1.00	1.10	0.088	0.991	0.344	0.780
Bradyrhizobiaceae	0.62	<0.01	0.46	0.55	0.72	0.17	0.019	0.493	0.774	0.662
Caulobacteraceae	0.52	0.71	1.02	1.25	0.71	0.75	0.777	0.414	0.236	0.756
Ruminococcaceae	0.50	0.54	0.57	1.05	1.23	0.44	0.930	0.863	0.205	0.095
Micrococcaceae	0.45	<0.01	0.60	1.53	0.88	0.40	0.460	0.791	0.055	0.440
Rhodobacteraceae	0.44	0.03	0.25	0.74	2.15	0.84	0.753	0.871	0.793	0.141
Brachyspiraceae	0.37	<0.01	0.03	2.59	0.05	1.21	0.843	0.836	0.180	0.848
Streptococcaceae	0.26	1.23	1.06	0.53	0.81	0.51	0.099	0.128	0.598	0.289
Bacillaceae	0.12	<0.01	1.15	0.23	1.14	0.53	0.877	0.159	0.886	0.163
Others	11.45	9.11	12.36	9.35	9.63	2.80	0.583	0.811	0.582	0.663

SPC = soy protein concentrate.

1 NC, PC, RFM, RPM, and RBP: basal diet without SPC (X-Soy 200, Selecta, MG, Brazil); basal diet with SPC replacing all animal protein supplements; basal diet with SPC replacing fish meal; basal diet with SPC replacing poultry meal; basal diet with SPC replacing blood plasma.

At genus level (Table 3), when SPC replaced poultry meal in the nursery diet, pigs tended to decrease (*P* = 0.064) the relative abundance of *Helicobacter* and tended to increase (*P* = 0.098) the relative abundance of *Methylobacterium*. When SPC replaced blood plasma in the nursery diet, pigs increased (*P* < 0.05) the relative abundance of *Cupriavidus*. When SPC replaced all animal protein supplements in the nursery diet, pigs increased (*P* < 0.05) the relative abundance of *Propionibacterium* and *Lactobacillus* and tended to increase the relative abundance of *Pelmonas* (*P* = 0.095), *Prevotella* (*P* = 0.077), and *Pseudomonas* (*P* = 0.090), whereas it decreased (*P* < 0.05) the relative abundance of *Helicobacter*, *Corynebacterium*, *Sphingomonas*, and *Facklamia* and tended to decrease (*P* = 0.084) the relative abundance of *Acinetobacter*.

**Table 3:** Relative abundance of jejunal mucosa-associated microbiota at genus level in nursery pigs fed diets with SPC replacing fish meal, poultry meal, and blood plasma.

Item	NC^1^	PC^1^	RFM^1^	RPM^1^	RBP^1^	SEM	*P*-value			
							NC vs. PC	NC vs. RFM	NC vs. RPM	NC vs. RBP
*Helicobacter*	31.03	8.01	25.76	13.10	22.13	6.99	0.034	0.580	0.064	0.352
*Pelomonas*	7.75	17.01	9.01	9.45	15.52	4.14	0.095	0.797	0.729	0.117
*Prevotella*	7.69	19.76	6.75	17.31	6.83	7.29	0.077	0.875	0.113	0.886
*Acinetobacter*	5.39	<0.01	1.53	2.68	1.52	2.09	0.084	0.164	0.326	0.163
*Clostridium*	3.93	5.15	5.50	5.41	3.10	3.35	0.789	0.701	0.717	0.840
*Corynebacterium*	3.37	<0.01	1.95	2.29	2.97	0.77	0.006	0.179	0.304	0.704
*Alcaligenes*	3.18	<0.01	0.16	0.01	<0.01	1.56	0.180	0.155	0.135	0.134
*Campylobacter*	2.90	0.19	1.36	4.13	0.93	1.80	0.304	0.513	0.599	0.404
*Staphylococcus*	2.81	0.08	2.99	5.00	4.69	1.27	0.160	0.913	0.206	0.277
*Microbacterium*	2.67	3.53	2.73	2.65	5.25	1.48	0.647	0.972	0.993	0.129
*Chlamydia*	2.04	5.25	0.24	0.10	0.08	2.06	0.274	0.493	0.460	0.453
*Pseudomonas*	1.68	8.06	3.71	1.62	2.20	2.72	0.090	0.541	0.986	0.876
*Propionibacterium*	1.58	5.73	3.69	1.23	2.65	1.80	0.040	0.236	0.847	0.546
*Sphingomonas*	1.18	0.01	1.08	0.80	1.25	0.38	0.046	0.842	0.465	0.897
*Bifidobacterium*	1.12	2.52	1.89	1.05	2.24	0.75	0.222	0.453	0.946	0.272
*Facklamia*	1.11	<0.01	0.43	0.65	0.35	0.36	0.046	0.166	0.341	0.123
*Cupriavidus*	1.11	0.58	1.34	1.31	2.40	0.50	0.439	0.705	0.752	0.041
*Methylobacterium*	0.95	0.63	1.53	2.13	2.09	0.62	0.682	0.414	0.098	0.111
*Lactobacillus*	0.94	8.93	4.35	4.70	3.19	2.14	0.017	0.245	0.200	0.439
*Ralstonia*	0.72	0.28	0.65	0.75	1.17	0.27	0.195	0.795	0.940	0.144
*Selenomonas*	0.70	0.08	0.30	1.58	0.18	0.52	0.436	0.575	0.225	0.462
*Succinivibrio*	0.69	2.78	0.68	1.77	1.01	1.21	0.101	0.992	0.338	0.775
*Mitsuokella*	0.62	0.56	0.91	1.14	1.15	0.41	0.929	0.603	0.359	0.349
*Arthrobacter*	0.41	<0.01	0.33	0.79	0.81	0.30	0.275	0.814	0.261	0.240
*Brevundimonas*	0.39	0.12	0.86	0.88	0.57	0.67	0.676	0.419	0.394	0.759
*Streptococcus*	0.28	1.48	1.33	0.64	0.95	0.63	0.105	0.114	0.591	0.311
*Bacillus*	0.10	<0.01	1.05	0.17	0.98	0.47	0.885	0.142	0.915	0.171
*Luteimonas*	<0.01	<0.01	3.07	<0.01	<0.01	1.49	1.000	0.134	1.000	0.998
Others	13.65	9.27	14.83	16.65	13.80	3.14	0.334	0.770	0.458	0.970

SPC = soy protein concentrate.

1 NC, PC, RFM, RPM, and RBP: basal diet without SPC (X-Soy 200, Selecta, MG, Brazil); basal diet with SPC replacing all animal protein supplements; basal diet with SPC replacing fish meal; basal diet with SPC replacing poultry meal; basal diet with SPC replacing blood plasma.

At species level (Table 4), when SPC replaced poultry meal in the nursery diet, pigs increased (*P* < 0.05) the relative abundance of *Prevotella stercorea* and decreased (*P* < 0.05) *Helicobacter rappini*. When SPC replaced blood plasma in the nursery diet, pigs increased (*P* < 0.05) the relative abundance of *Microbacterium ginsengisoli* and tended to increase (*P* = 0.098) the relative abundance of *Cupriavidus necator*. When SPC replaced all animal protein supplements in the nursery diet, pigs increased (*P* < 0.05) the relative abundance of *Prevotella copri*, *Propionibacterium acnes*, *Pelomonas aquatica*, and *Roseburia faecis* and tended to increase (*P* = 0.098) the relative abundance of *Bifidobacterium boum*, whereas it decreased (*P* < 0.05) the relative abundance of *H. rappini* and tended to decrease (*P* = 0.056) the relative abundance of *Facklamia ignava*.

**Table 4:** Relative abundance of jejunal mucosa-associated microbiota at species level in nursery pigs fed diets with SPC replacing fish meal, poultry meal, and blood plasma.

Item	NC^1^	PC^1^	RFM^1^	RPM^1^	RBP^1^	SEM	*P*-value			
							NC vs. PC	NC vs. RFM	NC vs. RPM	NC vs. RBP
*Helicobacter rappini*	27.36	4.27	15.57	7.11	16.06	5.26	0.006	0.104	0.006	0.119
*Prevotella copri*	8.60	24.81	8.61	18.28	9.15	8.45	0.035	0.999	0.155	0.936
*Pelomonas puraquae*	7.00	11.08	6.62	7.80	12.56	3.32	0.360	0.924	0.841	0.166
*Helicobacter mastomyrinus*	4.42	5.41	8.33	6.80	6.70	3.74	0.862	0.444	0.642	0.656
*Alcaligenes faecalis*	4.00	<0.01	0.21	0.01	<0.01	1.96	0.180	0.155	0.135	0.134
*Chlamydia suis*	2.94	6.80	0.37	0.16	0.14	2.75	0.322	0.461	0.426	0.422
*Microbacterium ginsengisoli*	2.65	2.46	2.84	2.75	5.64	1.35	0.904	0.894	0.949	0.042
*Propionibacterium acnes*	2.45	9.48	7.07	1.47	4.27	3.00	0.048	0.143	0.751	0.561
*Pelomonas aquatica*	2.14	6.40	3.23	2.99	4.56	1.54	0.027	0.517	0.613	0.153
*Facklamia ignava*	1.66	<0.01	0.59	1.12	0.62	0.56	0.056	0.166	0.476	0.175
*Helicobacter equorum*	1.65	1.33	0.34	0.27	0.38	0.68	0.762	0.164	0.143	0.177
*Clostridium butyricum*	1.19	1.12	1.19	1.75	0.86	0.97	0.959	0.997	0.662	0.793
*Cupriavidus necator*	0.91	1.02	1.02	0.99	1.82	0.45	0.860	0.845	0.882	0.098
*Corynebacterium imitans*	0.89	<0.01	0.79	0.67	0.87	0.42	0.169	0.871	0.709	0.981
*Roseburia faecis*	0.75	2.68	0.48	1.00	0.43	0.76	0.030	0.733	0.744	0.688
*Helicobacter* sp.	0.68	<0.01	3.49	0.41	<0.01	1.48	0.765	0.168	0.895	0.739
*Bifidobacterium boum*	0.62	1.84	0.92	0.40	1.27	0.50	0.098	0.646	0.735	0.321
*Prevotella stercorea*	0.56	2.21	0.56	2.86	0.92	1.08	0.107	0.995	0.014	0.690
*Succinivibrio dextrinosolvens*	0.55	0.30	0.18	1.28	0.80	0.72	0.747	0.605	0.312	0.733
*Campylobacter coli*	0.48	<0.01	0.14	4.38	0.13	2.01	0.876	0.901	0.158	0.900
*Mitsuokella jalaludinii*	0.44	0.28	0.58	0.51	0.89	0.28	0.703	0.713	0.850	0.250
*Prevotella ruminicola*	0.41	0.09	0.10	1.67	0.01	0.59	0.718	0.689	0.108	0.613
*Lactobacillus mucosae*	0.36	2.16	1.63	0.81	1.26	0.86	0.123	0.221	0.658	0.384
*Lactobacillus ruminis*	0.22	0.05	1.12	0.16	0.130	0.79	0.865	0.325	0.948	0.242
*Dialister succinatiphilus*	0.15	1.52	1.68	0.43	0.68	0.68	0.187	0.102	0.760	0.566
Others	26.93	14.51	32.34	33.92	28.68	5.33	0.145	0.475	0.356	0.816

SPC = soy protein concentrate.

1 NC, PC, RFM, RPM, and RBP: basal diet without SPC (X-Soy 200, Selecta, MG, Brazil); basal diet with SPC replacing all animal protein supplements; basal diet with SPC replacing fish meal; basal diet with SPC replacing poultry meal; basal diet with SPC replacing blood plasma.

**Fig. 4:**
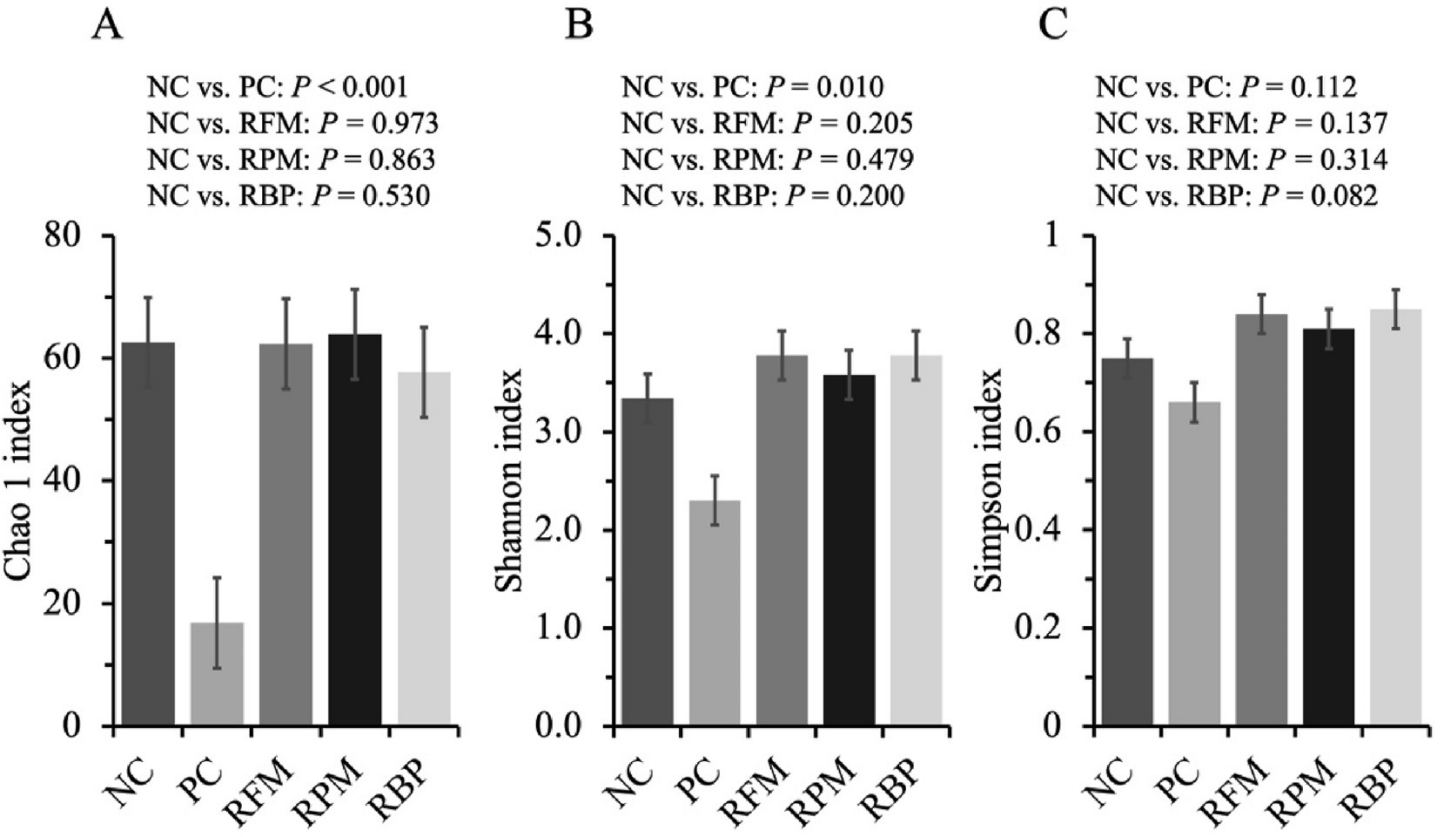
Alpha diversity of jejunal mucosa-associated microbiota at family level estimated with Chao1 richness (A), Shannon diversity (B), and Simpson diversity (C) in nursery pigs fed diets with SPC replacing fish meal, poultry meal, and blood plasma. Values are presented as mean ± standard error of the mean. Dietary treatments were supplemented with SPC (X-Soy 200, Selecta, MG, Brazil) replacing none (NC), all animal protein supplements (PC), fish meal (RFM), poultry meal (RPM), or blood plasma (RBP) in nursery diets. SPC = soy protein concentrate.

**Fig. 5:**
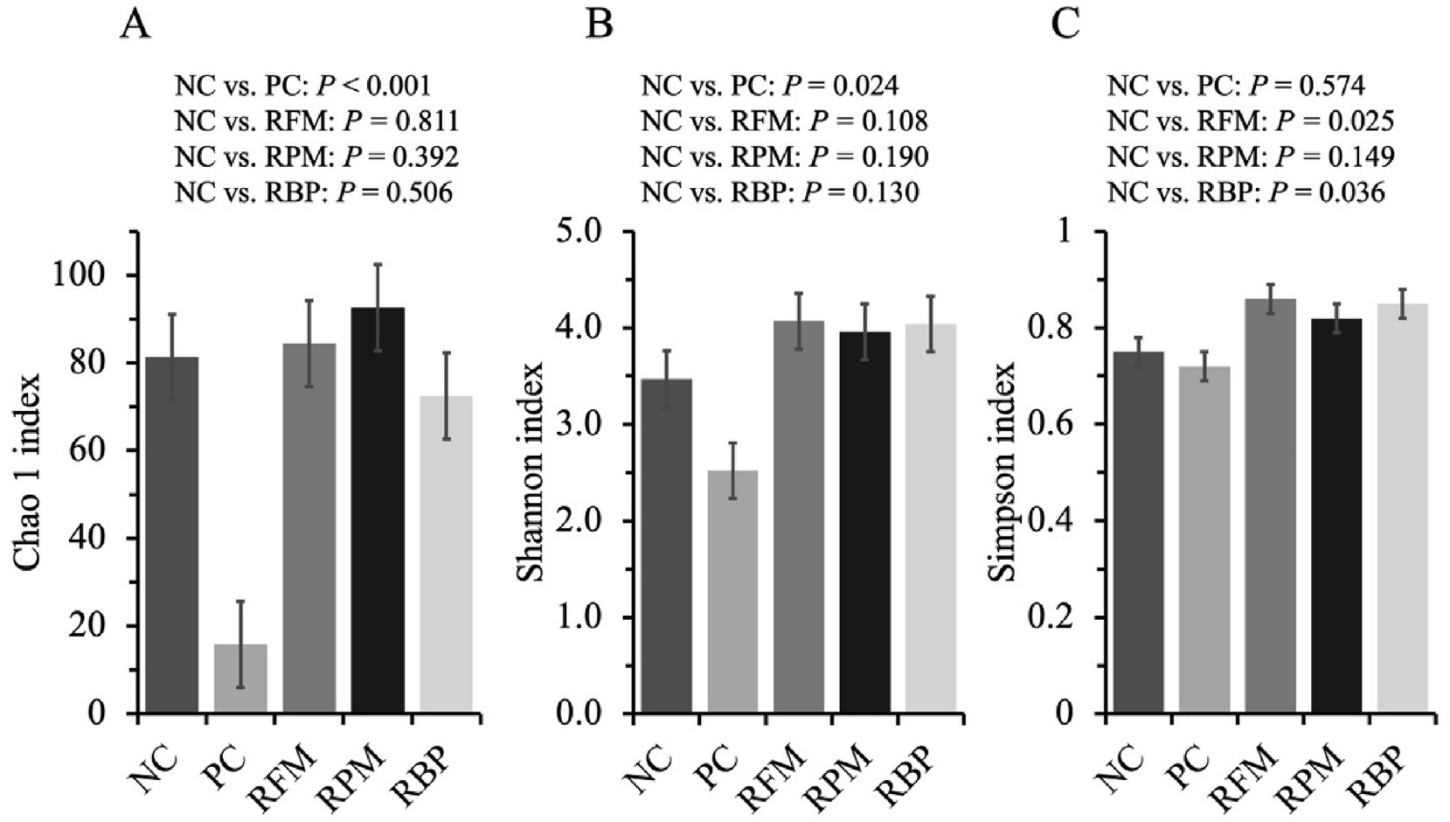
Alpha diversity of jejunal mucosa-associated microbiota at species level estimated with Chao1 richness (A), Shannon diversity (B), and Simpson diversity (C) in nursery pigs fed diets with SPC replacing fish meal, poultry meal, and blood plasma. Values are presented as mean ± standard error of the mean. Dietary treatments were supplemented with SPC (X-Soy 200, Selecta, MG, Brazil) replacing none (NC), all animal protein supplements (PC), fish meal (RFM), poultry meal (RPM), or blood plasma (RBP) in nursery diets. SPC = soy protein concentrate.

For alpha diversity, when SPC replaced blood plasma in the nursery diet, pigs tended to increase Simpson index at family (*P* = 0.082) and increased (*P* < 0.05) at species level (Fig. 4, Fig. 5). When SPC replaced fish meal in the nursery diet, pigs increased (*P* < 0.05) the Simpson index at species level. When SPC replaced all animal protein supplements in the nursery diet, pigs decreased (*P* < 0.05) Shannon and Chao 1 indexes at family and species levels.

### 3.3 Immune and oxidative stress status

When SPC replaced fish meal in the nursery diet, pigs tended to increase (*P* = 0.096) the IgA content in the jejunal mucosa compared to NC (Table 5). The concentration of TNF-α, IL-8, IgG, protein carbonyl, and MDA were not affected by SPC replacing animal protein supplements.

**Table 5:** Oxidative stress and immune parameters of nursery pigs fed diets with SPC replacing all animal protein supplements, fish meal, poultry meal, and blood plasma.

Item	NC^1^	PC^1^	RFM^1^	RPM^1^	RBP^1^	SEM	*P*-value			
							NC vs. PC	NC vs. RFM	NC vs. RPM	NC vs. RBP
Jejunal mucosa										
TNF-α,^2^ pg/mg of protein	0.32	0.30	0.42	0.34	0.37	0.06	0.788	0.240	0.835	0.526
IL-8,^3^ pg/mg of protein	339	339	335	313	343	39	0.993	0.929	0.589	0.938
IgA,^4^ μg/mg of protein	3.48	5.54	5.54	2.99	4.66	0.90	0.126	0.096	0.678	0.325
IgG,^5^ μg/mg of protein	2.62	2.52	3.51	2.36	3.21	0.52	0.897	0.209	0.700	0.391
MDA, nmol/mg of protein	0.86	0.83	0.74	0.91	0.68	0.11	0.810	0.397	0.746	0.219
Protein carbonyl, nmol/mg of protein	1.28	1.14	1.68	1.26	1.57	0.41	0.654	0.160	0.942	0.271

SPC = soy protein concentrate.

1 NC, PC, RFM, RPM, and RBP: basal diet without SPC (X-Soy 200, Selecta, MG, Brazil); basal diet with SPC replacing all animal protein supplements; basal diet with SPC replacing fish meal; basal diet with SPC replacing poultry meal; basal diet with SPC replacing blood plasma.

2 TNF-α, tumor necrosis factor alpha.

3 IL-8, interleukin 8.

4 IgA, immunoglobulin A.

5 IgG, immunoglobulin G.

### 3.4 Intestinal morphology and crypt cell proliferation

**Fig. 6:**
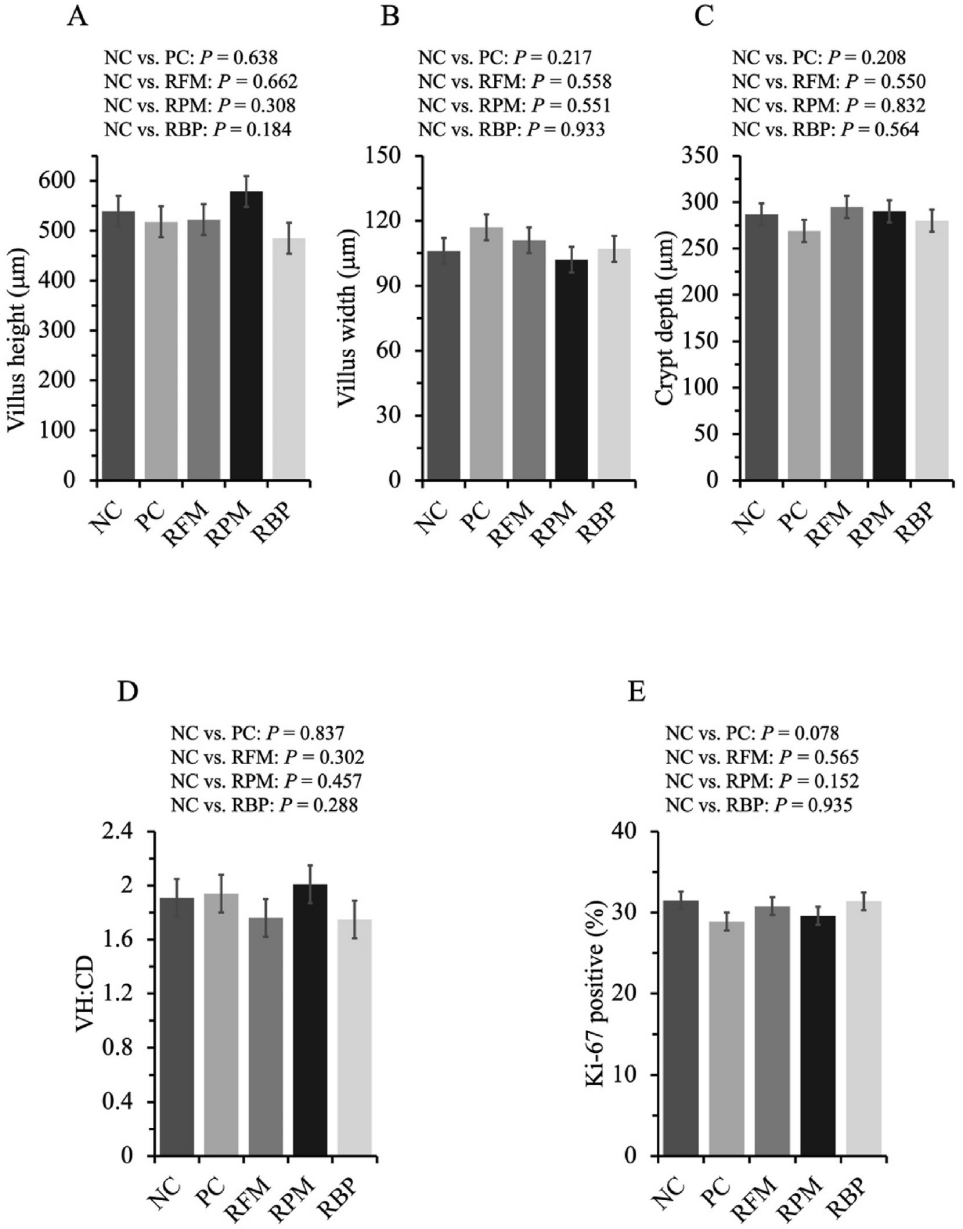
Jejunal morphology and the ratio of Ki-67 positive cells to total cells in the crypt of nursery pigs fed diets with SPC replacing all animal protein supplements, fish meal, poultry meal, and blood plasma. (A) Villus height of jejunum. (B) Villus width of jejunum. (C) Crypt depth of jejunum. (D) Villus height to crypt depth ratio. (E) Crypt cell proliferation rate. Values are presented as mean ± standard error of the mean. Dietary treatments were supplemented with SPC (X-Soy 200, Selecta, MG, Brazil) replacing none (NC), all animal protein supplements (PC), fish meal (RFM), poultry meal (RPM), or blood plasma (RBP) in nursery diets. SPC = soy protein concentrate.

When SPC replaced all animal protein supplements in the nursery diet, pigs tended to decrease (*P* = 0.078) the ratio of Ki-67 positive cells to total cells in the crypt (Fig. 6). The villus height, villus width, crypt depth, and VH:CD ratio were not affected by SPC replacing animal protein supplements.

### 3.5 Apparent ileal digestibility

When SPC replaced all animal protein supplements in the nursery diet, the AID of DM, GE, EE, CP, and essential AA were not affected among treatments (Table 6).

**Table 6:** Apparent ileal digestibility of nursery pigs fed diets with SPC replacing all animal protein supplements, fish meal, poultry meal, and blood plasma (%).

Item^1^	NC^2^	PC^2^	RFM^2^	RPM^2^	RBP^2^	SEM	*P*-value			
							NC vs. PC	NC vs. RFM	NC vs. RPM	NC vs. RBP
DM	53.2	61.6	54.5	60.3	58.0	3.9	0.163	0.796	0.191	0.371
GE	52.6	58.4	48.4	54.0	54.2	4.8	0.335	0.451	0.803	0.772
EE	72.2	74.9	68.4	67.4	74.5	6.1	0.682	0.529	0.405	0.690
CP	62.2	69.8	58.2	66.5	62.1	3.9	0.187	0.418	0.393	0.985
Lys	72.6	79.2	70.7	74.0	71.9	4.9	0.241	0.688	0.769	0.872
Met + Cys	63.0	72.4	59.5	68.8	64.8	4.2	0.115	0.497	0.265	0.726
Trp	67.2	73.7	62.7	70.6	70.3	4.3	0.277	0.389	0.517	0.551
Thr	60.4	65.4	52.1	63.9	60.1	4.5	0.442	0.146	0.546	0.962
Val	61.8	68.5	55.3	63.0	61.7	4.0	0.254	0.197	0.815	0.977
Ile	66.4	73.1	60.8	67.8	66.2	3.3	0.169	0.189	0.729	0.970
Leu	63.0	71.5	60.7	67.4	64.6	3.7	0.118	0.612	0.351	0.725
Phe	66.2	73.9	63.0	70.2	67.3	3.2	0.108	0.433	0.342	0.782
His	67.6	74.1	64.0	71.0	67.3	3.2	0.155	0.357	0.404	0.941
Arg	77.3	82.1	75.0	80.4	76.8	2.6	0.168	0.430	0.318	0.850

SPC = soy protein concentrate; DM = dry matter; GE = gross energy; EE = ether extract; CP = crude protein.

1 The digesta samples were collected at the end of experiment (d 35).

2 NC, PC, RFM, RPM, and RBP: basal diet without SPC (X-Soy 200, Selecta, MG, Brazil); basal diet with SPC replacing all animal protein supplements; basal diet with SPC replacing fish meal; basal diet with SPC replacing poultry meal; basal diet with SPC replacing blood plasma.

## 4 Discussion

The use of soybean meal is limited in diets for nursery pigs due to the content of anti-nutritional factors including allergenic proteins (Dunsford et al., 1989; Li et al., 1990; Taliercio et al., 2014). Soy allergenic proteins can result in an immune disorder in nursery pigs. Sun et al. (2008) indicated that glycinin-treated pigs could increase their immune responses in jejunal mucosa with increased levels of IgA, IL-4, and IL-6, however IgG and IgM were not affected. In addition, β-conglycinin has been demonstrated to increase the concentrations of IgA, IL-2, IL-6, and interferon-γ, in peripheral blood mononuclear cells of suckling pigs (Jun et al., 2009). Animal protein supplements, including blood plasma, fish meal, and poultry meal, are regularly used in diets for pigs during nursery period to reduce the use of soybean meal because they contain high quality protein content and are free of anti-nutritional factors and allergens. In this study, supplementation of SPC replacing animal protein supplements did not affect the overall immune and oxidative status in the jejunal mucosa, and fecal score of nursery pigs. These results can be related to the low amount of soy antigens in SPC including glycinin and β-conglycinin that are significantly decreased after ethanol extraction (Peisker, 2001). In addition, SPC supplementation replacing fish meal, poultry meal or blood plasma in nursery diets did not affect the growth performance of pigs during the overall experimental period. However, the complete replacement of animal protein supplements by SPC reduced the daily gain by reducing the feed intake of pigs. Lenehan et al. (2007) indicated that high level of SPC in nursery diets may affect the palatability, thus decreasing the feed intake, which was in agreement with the result of the current study. In addition, Deng et al. (2022) reported that SPC supplementation can increase the anorectic hormone, which caused reduced feed intake of nursery pigs.

Fish meal is commonly used in diets for nursery pigs due to its high protein content with a desirable AA profile. Previous study indicated that feeding fish meal in the nursery diet can improve the growth performance of pigs (Kim and Easter, 2001). According to Calder (2001), the fatty acids in fish meal, such as eicosapentaenoic acid and docosahexaenoic acid, had been indicated to decrease proinflammatory cytokines.

Poultry meal, as a by-product derived from the poultry slaughtering process, has been shown as a high quality protein supplement that can efficiently replace fish meal without influencing growth performance of pigs during nursery period (Keegan et al., 2004). However, the composition and protein digestibility of poultry meal are variable because of the different raw materials and manufacturing processes (Dong et al., 1993). In the current study, when poultry meal was replaced by SPC, no difference in growth performance, intestinal immune and oxidative stress status were observed.

Blood plasma, as a high-quality protein supplement, has been demonstrated to enhance the growth performance of pigs during the first week after weaning (van Dijk et al., 2001). In addition, the immunoglobulin fractions in the blood plasma play a pivotal role in immune protection and prevention of pathogen adhesion by blocking the receptor-binding site of adhesion bacteria (Nollet et al., 1999). In this study, the supplementation of SPC replacing blood plasma in nursery diet reduced feed intake and ADG in P1, which might be attributed to the removal of benefits of blood plasma for nursery pigs. Previous study has indicated that the supplementation of blood plasma can enhance the feed intake of newly-weaned pigs, mainly during the first week after weaning (Pierce et al., 2005).

In this study, in order to evaluate the intestinal health of nursery pigs, jejunal samples were collected at the end of the experiment. Although the protein levels were not consistent among phases, previous studies indicated that the changes of the intestinal environment caused by dietary intervention have a long-lasting effect on intestinal health of nursery pigs (Deng et al., 2022; Duarte and Kim, 2022b). In addition, the hypothesis of this study was to evaluate the effect of SPC replacing animal protein supplements during the entire nursery period. Pigs were individually housed to assess the effects of accurate SPC intake on the growth performance, immune status, oxidative stress, intestinal morphology, nutrient digestibility, and mucosa-associated microbiota of nursery pigs, in accordance with previous studies (Jang and Kim, 2019; Moita et al., 2022). However, it is important to note that environmental factors such as housing conditions, feed interactions, and age may influence the impact of SPC on intestinal oxidative stress, immune status, morphology, and nutrient digestibility. Previous studies have indicated that group housing of pigs may influence their physiological responses, behavior, as well as intestinal immune response and microbiota composition (Bruininx et al., 2002; Wen et al., 2021).

The soybean antigens can affect the intestinal morphology reflecting the capacity for digestion and absorption of nutrients. According to Qin et al. (2002), antigens in soybean induced allergies that can decrease villus height and increase crypt depth in pigs. In the current study, the unchanged intestinal morphology among treatments could be associated with the low concentration of antigens in SPC. A lower crypt cell proliferation was observed when SPC replaced all animal protein supplements. According to Jang and Kim (2019), the concentration of nucleotides in animal protein supplements was greater than in plant protein supplements. Crypt cell proliferation in pigs can be increased with the supplementation of nucleotides (Domeneghini et al., 2004). In the current study, 18%, 12%, and 6% animal protein supplements were replaced in phases 1, 2, and 3 by SPC in PC treatment, which could result in consistently low amounts of nucleotides in PC treatment that may negatively affect crypt cell proliferation. In addition, blood plasma has been indicated to improve the proliferation of enterocytes in nursery pigs (Tran et al., 2014). Therefore, when SPC was supplemented replacing all animal protein supplements, the reduction of exogenous nucleotides and blood plasma in the diet could be the potential reason for decreased crypt cell proliferation.

The difference in dietary protein sources has been suggested that can modulate the microbiota composition in the intestine of pigs (Rist et al., 2013). In this study, decreased Proteobacteria and increased Bacteroidetes were observed when poultry meal or all animal protein supplements were replaced by SPC. It can be speculated that the removal of poultry meal in nursery diets could result in these changes. Previous studies indicated that poultry meal may have the lower digestibility compared with other animal protein supplements due to the various raw material (Moser et al., 1998; Orozco-Hernandez et al., 2003). When poultry meal was included in nursery diets, the undigested proteins from poultry meal might increase the nitrogen availability and the pH of the digesta in the intestine, which provides a favorable environment for pathogens and proteolytic bacteria (Kim et al., 2018). However, the nutrient digestibility was not affected by the treatments in this study. The reduction of Proteobacteria mainly resulted from the decrease in family Helicobacteraceae and genus *Helicobacter*, which has been correlated to unhealthy status (Duarte and Kim, 2022a; Zhang et al., 2017). However, the increased Bacteroidetes resulting from increased Prevotellaceae has been related to improved health of pigs (Crespo-Piazuelo et al., 2018; Duarte et al., 2021). Interestingly, the relative abundance of Enterobacteriaceae increased when SPC replaced all animal protein supplements. This result can be associated with the reduction in dietary nucleotide when all the animal protein supplements were replaced in the diet (Jang and Kim, 2019). Uauy et al. (1994) indicated that dietary nucleotides can stimulate the growth of favorable bacteria, however, this may be at the cost of a reduction of Enterobacteriaceae. Additionally, the immunoglobulins in the blood plasma have been associated with decreased Proteobacteria in the intestine of rats and pigs (Balan et al., 2013; Cheng et al., 2021). Although the abundance of Enterobacteriaceae was increased, the abundance of Proteobacteria decreased when SPC replaced all animal protein supplements, which was mainly related to the decrease of Helicobacteraceae. In addition, several studies have been conducted to evaluate the effects of different protein sources on intestinal microbiota. According to Iakhno et al. (2020), yeast replacing 40% of conventional protein can change the composition of microbiota in the intestinal digesta of nursery pigs. Cheng et al. (2021) showed similar microbiota changes as in this study when the animal proteins were replaced by *Corynebacterium glutamicum* cell mass. Furthermore, the protein level in diets can be another factor to affect the intestinal microbiota and their metabolites. Chen et al. (2018) reported that reducing the dietary protein from 18% to 15% decreased the relative abundance of potentially harmful bacteria and increased beneficial bacteria, such as *Bifidobacterium*, and *Lactobacillus* in ileal digesta of growing pigs. Previous study also indicated that high SPC inclusion level replacing animal protein supplements could cause similar shift in jejunal mucosa-associated microbiota (Deng et al., 2022). In this study, the protein level of PC and RPM treatments were relatively lower than other treatments and both treatments have higher SPC inclusion level, which could be the potential reasons causing the shift of intestinal microbiota.

Even though the changes in relative abundance of microbiota followed a similar trend when SPC replaced poultry meal or all animal protein supplements, the alpha diversity only decreased in PC treatment. Higher microbial diversity has been related with improved health in pigs and reduced susceptibility of allergic diseases due to their function to modulate the immune responses (Looft et al., 2014; Ober et al., 2017; Vo et al., 2017; Xu et al., 2022). Furthermore, diverse intestinal microbiota can provide many beneficial functions, including the production of vitamins, volatile fatty acids, and fiber digestion (Kim and Isaacson, 2015). In the current study, the reduced diet complexity in PC treatment could be the potential reason for the reduced alpha diversity of microbiota. Previous study indicated that including more feed ingredients in diets could be a strategy to increase diversity of intestinal microbiota in pigs, thus preventing the incidence of diarrhea and the use of antibiotics (Fouhse et al., 2016).

## 5 Conclusion

In conclusion, the current study demonstrated SPC could replace fish meal, poultry meal, or blood plasma in diets of nursery pigs without affecting growth performance, overall intestinal health, and nutrient digestibility. Particularly, SPC replacing poultry meal in nursery diets enhanced the balance of jejunal mucosa-associated microbiota by reducing *H. rappini* and increasing *P. stercorea*. However, replacing all fish meal, poultry meal, and blood plasma by SPC reduced ADG of nursery pigs mainly due to the reduction of voluntary feed intake.

## Author contributions

**Sung Woo Kim:** Conceptualization, Methodology, Investigation, Validation, Resources, Writing - Review & Editing, Supervision, Project administration, Funding acquisition. **Zixiao Deng:** Methodology, Formal analysis, Investigation, Data Curation, Writing - Original Draft, Visualization. **Marcos Elias Duarte:** Methodology, Formal analysis, Investigation, Data Curation, Writing - Writing - Review & Editing, Visualization.

## Declaration of competing interest

We declare that we have no financial and personal relationships with other people or organizations that can inappropriately influence our work, and there is no professional or other personal interest of any nature or kind in any product, service and/or company that could be construed as influencing the content of this paper.
